# Supplementary material for: Happy work: Improving enterprise human resource management by predicting workers’ stress using deep learning
Source: PLoS One. 2022 Apr 13;17(4):e0266373. doi: 10.1371/journal.pone.0266373 (PMC9007354; doi:10.1371/journal.pone.0266373)
Supplement: Attachment — Submitted filename: Response_letter.pdf [file pone.0266373.s003.pdf]

Response to Reviewers' Comments

Happy Work: Improving Enterprise Human Resource Management by Predicting Workers' Stress using Deep Learning

We would like to thank the editor and the reviewers for their careful reading and valuable comments that have helped us to improve this manuscript. We have made several improvements to the manuscript following the reviewers' comments and suggestions. In the marked-up revision, we highlighted the added changes in blue and the deleted changes in red for the reviewers' convenience. Now we first summarize these main changes and then respond to each of the reviewer's comments in detail.

1. We have discussed the difference between our work and the questionnaire-based stress level detection (Work Stress Questionnaire, WSQ [1]).

2. We have added three most related works and described their difference from our work to highlight the significance.

3. We have corrected the figures, typos and confusions according to reviewers' comments.

The detailed responses to each comment are outlined next. All comments from reviewers are reproduced in *italic*.

1 Responses to Reviewer #1's Comments:

1. *The idea of detecting stress level based on factual data rather than subjective data with ANNs is novel. However, the manuscript should be organized better to support this proposal.*

   Response: Thanks for appreciating our idea. We will update and organize better the manuscript to support this proposal according to your suggestions.

2. *The work proposes detecting stress level of workers based on objective worker's data would deliver better results than a potentially subjective questionnaire. After that it conducts experiments on some objective data of workers and finds accuracy results. However, merely delivering this result doesn't support its claimed novelty; therefore, a comparison between stress level detection accuracy based on a questionnaire and based on proposed method should be illustrated.*

   Response: Thanks for the suggestion to make comparison with the questionnaire-based stress level detection, in order to highlight our novelty. We would like to explain this point as follows. Actually, we propose the artificial neural networks (ANN)-based stress level detection approach and apply this approach to any objective worker's data, including but not limited to salary, working time, KPI. Therefore, our approach is more general due to its objective, quantitative and automated property. Once our approach trains a general prediction model based on the collected data, it can be adaptive to predict the new instances (i.e., workers). In contrast, the questionnaire-based stress level detection (Work Stress Questionnaire, WSQ [1]) is more specific and lacks adaptation to new instances. Besides, it requires tedious manual effort to collect data and analyze data. Therefore, it is difficult to directly compare them in experiments and obtain an empirical result. We summarize the difference of them in a table below.

Table 1: Comparing our method with questionnaire-based stress level detection (WSQ).

**Ours**	**WSQ [1]**
General	Specific
Adaptive	Static
Quantitative	Qualitative
Automatic	Manual

   It should be noted that our method can be extended to handle the dataset collected from questionnaire. That is why our method is more general than the questionnaire-based stress level detection methods.

   We have added the above discussion into the updated version. We hope that the Reviewer is now satisfied by the modifications applied to this revision.

3. *The performance of proposed model (using ANNs for classification) may be compared to performance of other non-SOTA ML classifiers (i.e. SVM) to*

see its contribution.

Response: Thanks for pointing this out. We agree with your comment on this point. According to your suggestion, we have used SVM as baseline and added the comparison result about the stress classification model. The new result is shown in the following Table.

Table 2: Comparing our stress classification model with SVM classifier.

Methods	Ours	SVM
**Accuracy**	71.2%	60.9%
**F1 score**	68.6	57.9

From Table 2, we can see that the accuracy and F1 score metrics of our stress classification model are 71.2% and 68.6, respectively. The accuracy and F1 score metrics of the SVM-based stress classifier are 60.9% and 57.9, respectively. Therefore, our stress classification model outperforms the SVM-based stress classifier when predicting workers' stress statuses.

We have incorporated the new result in the updated version. We hope that the Reviewer is now satisfied by the modifications applied to this revision.

4. *Access links of the datasets are not available via Kaggle but they are attached to the manuscript. Therefore, it is not clear that these are existing sets that developed by others or newly developed by the authors. If the data is developed by the authors, then data generation process should be illustrated. The Figure 2 gives steps about data collection and preprocessing, the manuscript should also detail these steps.*

   Response: The datasets (ESI and HAJP) we use are public in Kaggle. ESI is available in:

   `https://www.kaggle.com/mohamedharris/employee-satisfaction-index-dataset`

   while HAJP is available in:

   `https://www.kaggle.com/mfaisalqureshi/hr-analytics-and-job-prediction.`

   We will add the above links in the updated version. We hope that the Reviewer is now satisfied by the modifications applied to this revision.

5. *In Figure 5, the confusion matrices are given based on percentages of total number of workers in ESI dataset (i.e., 500 workers). When the given percentages are multiplied with the total worker number, the sum doesn't end up to 500. In other words, the percentages do not sum up to 1. Therefore, it is not possible to compute the accuracy metric from the confusion matrix.*

Response: Thanks for pointing this out. We agree with your comment on this point. According to your comment, we have found the error in computation. We have corrected the new results and presented the new figures in the updated version.

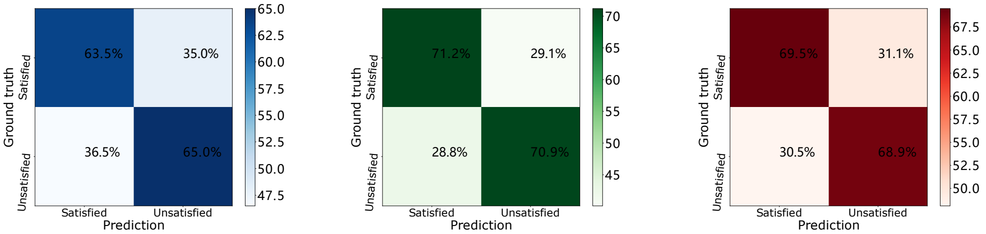

(a) training phase (b) validation phase (c) testing phase

Figure 1: Confusion matrices of our DNN model over ESI dataset.

6. *In Figure 2, testing process is shown as a distinct process then the training process. The validation process is not shown in the figure; however, it is expected to be part of the training process. The accuracy metric of the manuscript is shown as performance of the validation process, which is not expected to be. The overall performance of the network should be indicated as performance of a distinct testing process on never-seen data. This should clearly be illustrated in the Figure 2 and results should be organized accordingly.*

   Response: Thanks for pointing this out. 1) We agree with your comment on Figure 2. According to your suggestion, we have updated Figure 2. The new figure is shown below. 2) We report the test results during the training process because it is convenient to illustrate the test performance. Otherwise, the test performance becomes a point instead of a curve. Note that the test data was never used during the training process in the experiments.

   We have clearly updated the manuscript according to your suggestions. We hope that the Reviewer is now satisfied by the modifications applied to this revision.

7. *The manuscript includes some capitalization and grammatical errors.*

   Response: Thanks for the comment. We have corrected the capitalization and grammatical errors in the updated version. We hope that the Reviewer is now satisfied by the modifications applied to this revision.

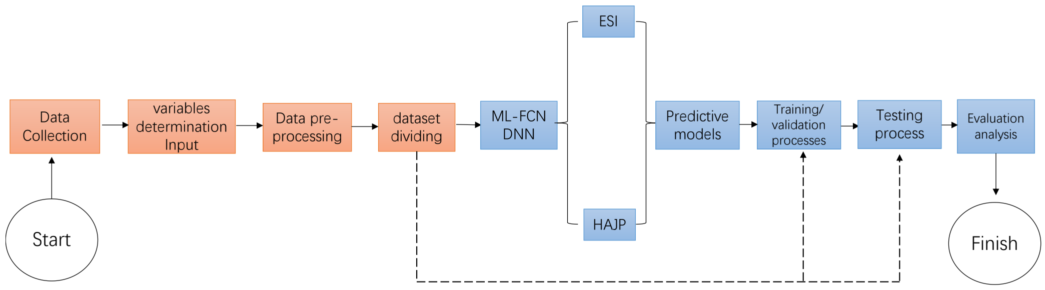

Figure 2: The procedure of constructing a predictive model.

8. *In the Related Works section, the general review of ANN's is given lengthy compared to works related to stress level detection methods. When a quick search is conducted, it is possible to find various methods for stress detection with ANN based on different type of data. A couple of them are:*
`https://www.mdpi.com/2227-7390/9/6/626/htm`
`https://bmcmedinformdecismak.biomedcentral.com/articles/10.1186/s12911-020-01299-4`
`https://ieeexplore.ieee.org/abstract/document/8834624`

Response: Thanks for suggesting important references. We have added and discussed the suggested references in the related work. Our discussion about this point is given as follows.

The existing works [2–4] are most related to our method. Umematsu *et al.* [2] study students' daily life stress forecasting based on the long short-term memory (LSTM) neural network and students' data (i.e., physiological signals, mobile phone usage, location, and behavioral surveys). Differently, we study the workers' stress prediction problem in the context of human resource management and use the enterprise-related datasets. Li *et al.* [3] study stress detection using 1-dimensional (1D) convolutional neural network and a multilayer perceptron neural network. Note that the dataset they use is physiological data collected from chest-worn and wrist-worn sensors. Collecting the physiological data from workers is difficult due to requiring the workers to wear the handset sensors. Bekesiene *et al.* [4] propose an ANN-based framework to predicate the stress level of military conscripts.

However, they use the military data, which is secret. Besides, they do not study the stress regression model, which is one of focus in our work.

We have added the above content in the updated version. We hope that the Reviewer is now satisfied by the modifications applied to this revision.

9. *This statement is not tested, or results are not illustrated: "The model performance can be improved when the size of the training dataset is increased and the samples are heterogeneous."*

   Response: Thanks for pointing this out. Our description here may be inaccurate. However, there is no doubt that the data distribution and the size of dataset affect the model performance (i.e., loss and accuracy). In fact, ther are also some existing works [5, 6] supporting this claim. When the data samples are more diverse and heterogeneous, the data distribution in the dataset is more approximate to the original ground-truth distribution. Thus, the trained model can learn more and have more powerful prediction. And when the size of dataset is large, the dataset is more likely diverse and heterogeneous. However, the impact of dataset distribution is more significant than the one of dataset size [6].

   We have correspondingly updated the statement in the revision. We hope that the Reviewer is now satisfied by the modifications applied to this revision.

10. *In Figure 3, the given training, validation, and test set graphs imply that they are generated during the training process. In other words, the test data appears to be used in the training process, however, it is possible that the corresponding intermediate results on the test data did not contribute to the training process contrary to validation results. If this is the case (i.e. test data is tested on the intermediate trained model but the results were not used to improve the intermediate model), then it should clearly be indicated in the manuscript. If the test data was never used during the training, then the figures should be explained accordingly.*

    Response: Thanks for pointing this out. We would like to explain this point as follows. We report the test results during the training process because it is convenient to illustrate the test performance. Otherwise, the test performance becomes a point instead of a curve. Note that we split the datasets into training datasets, validating datasets, and testing datasets such that they have no intersections. Thus, the test data was never used during the training

process. To eliminate the confusion in this point, we change the label of x-axis to be "epoch".

We agree that this illustrative way is not the best way to demonstrate our result. We have clarified this point in the updated version according to your suggestion.

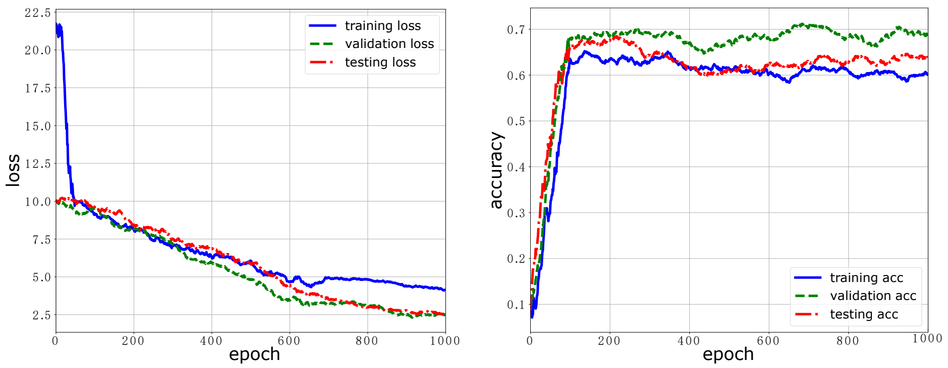

(a) model loss (b) model accuracy

Figure 3: The loss and accuracy performances of our DNN model over ESI dataset.

2 Responses to Reviewer #2's Comments:

1. *It is recommended to add the structure of the paper at the end of introduction section.*

   Response:

   Thanks for the comment. We have added the organization of the paper at the end of introduction section as suggested.

2. *Contribution 2 is not clear which is the main contribution of this study author should explain it in details.*

   Response:

   Thanks for the comment. We have given more explanations about contribution 2 in in details. We have also highlightd the key points about contribution in the updated version. We hope that the Reviewer is now satisfied by the modifications applied to this revision.

3. *The results are not discussed, need to discus results critically.*

   Response:

   Thanks for the comment. We have discussed the results critically in our updated version as suggested. We hope that the Reviewer is now satisfied by the modifications applied to this revision.

4. *Author could use the recent publish work in Deep Learning application such as* `https://journal.esj.edu.iq/index.php/IJCM/article/view/100`.

   Response:

   Thanks for the comment. We have incorporated the deep learning reference [7] as suggested in our updated version.

3 Responses to Reviewer #3's Comments:

1. *Thanks to the authors for this research effort and the idea that contributes to promoting an intelligent, AI-based employee management approach, specifically deep learning, away from traditional methods. The research is interesting in its idea, implementation and depiction of its results.*

   Response:

   Thanks for appreciating our work.

4 Conclusion

We would like to take this opportunity to express our appreciation for your expertise and invaluable assistance in reviewing our draft. We hope that the revised draft provides you a better presentation of our work. Should you have any questions, please do not hesitate to contact us.

Respectfully yours,

Yu Zhang, Ershi Qi

References

[1] A. Frantz and K. Holmgren, "The work stress questionnaire (wsq)–reliability and face validity among male workers," *BMC public health*, vol. 19, no. 1, pp. 1–8, 2019.

[2] T. Umematsu, A. Sano, S. Taylor, and R. W. Picard, "Improving students' daily life stress forecasting using lstm neural networks," in *2019 IEEE EMBS International Conference on Biomedical & Health Informatics (BHI)*. IEEE, 2019, pp. 1–4.

[3] R. Li and Z. Liu, "Stress detection using deep neural networks," *BMC Medical Informatics and Decision Making*, vol. 20, no. 11, pp. 1–10, 2020.

[4] S. Bekesiene, R. Smaliukiene, and R. Vaicaitiene, "Using artificial neural networks in predicting the level of stress among military conscripts," *Mathematics*, vol. 9, no. 6, p. 626, 2021.

[5] J. Prusa, T. M. Khoshgoftaar, and N. Seliya, "The effect of dataset size on training tweet sentiment classifiers," in *2015 IEEE 14th International Conference on Machine Learning and Applications (ICMLA)*. IEEE, 2015, pp. 96–102.

[6] A. Althnian, D. AlSaeed, H. Al-Baity, A. Samha, A. B. Dris, N. Alzakari, A. Abou Elwafa, and H. Kurdi, "Impact of dataset size on classification performance: an empirical evaluation in the medical domain," *Applied Sciences*, vol. 11, no. 2, p. 796, 2021.

[7] K. Aggarwal, M. M. Mijwil, A.-H. Al-Mistarehi, S. Alomari, M. Gök, A. M. Z. Alaabdin, S. H. Abdulrhman *et al.*, "Has the future started? the current growth of artificial intelligence, machine learning, and deep learning," *Iraqi Journal For Computer Science and Mathematics*, vol. 3, no. 1, pp. 115–123, 2022.
